# Transcriptomics and metabolomics analysis of L-phenylalanine overproduction in *Escherichia coli*

**DOI:** 10.1186/s12934-023-02070-w

**Published:** 2023-04-06

**Authors:** Wei Sun, Dongqin Ding, Danyang Bai, Yang Lin, Yaru Zhu, Cuiying Zhang, Dawei Zhang

**Affiliations:** 1 School of Biological Engineering, Tianjin University of Science and Technology, Tianjin, 300457 China; 2 Tianjin Institute of Industrial Biotechnology, Chinese Academy of Sciences, Tianjin, 300308 China; 3 National Technology Innovation Center of Synthetic Biology, Tianjin, 300308 China; 4 University of Chinese Academy of Sciences, Beijing, 100049 China

**Keywords:** *Escherichia coli*, L-phenylalanine production, Transcriptomic analysis, Metabolomic analysis, Metabolic flux

## Abstract

**Background:**

Highly efficient production of L-phenylalanine (L-Phe) in *E. coli* has been achieved by multiple rounds of random mutagenesis and modification of key genes of the shikimate (SHIK) and L-Phe branch pathways. In this study, we performed transcriptomic (16, 24 and 48 h) and metabolomic analyses (8, 16, 24, 32,40, and 48 h) based on time sequences in an engineered *E. coli* strain producing L-Phe, aiming to reveal the overall changes of metabolic activities during the fermentation process.

**Results:**

The largest biomass increase rate and the highest production rate were seen at 16 h and 24 h of fermentation, respectively reaching 5.9 h^−1^ and 2.76 g/L/h, while the maximal L-Phe titer of 60 g/L was accumulated after 48 h of fermentation. The DEGs and metabolites involved in the EMP, PP, TCA, SHIIK and L-Phe-branch pathways showed significant differences at different stages of fermentation. Specifically, the significant upregulation of genes encoding rate-limiting enzymes (*aroD* and *yidB*) and key genes (*aroF*, *pheA* and *aspC*) pushed more carbon flux toward the L-Phe synthesis. The RIA changes of a number of important metabolites (DAHP, DHS, DHQ, Glu and PPN) enabled the adequate supply of precursors for high-yield L-Phe production. In addition, other genes related to Glc transport and phosphate metabolism increased the absorption of Glc and contributed to rerouting the carbon flux into the L-Phe-branch.

**Conclusions:**

Transcriptomic and metabolomic analyses of an L-Phe overproducing strain of *E. coli* confirmed that precursor supply was not a major limiting factor in this strain, whereas the rational distribution of metabolic fluxes was achieved by redistributing the carbon flux (for example, the expression intensity of the genes *tyrB*, *aspC*, *aroL* and *aroF*/*G*/*H* or the activity of these enzymes is increased to some extent), which is the optimal strategy for enhancing L-Phe production.

**Supplementary Information:**

The online version contains supplementary material available at 10.1186/s12934-023-02070-w.

## Background

L-Phenylalanine (L-Phe) is an important aromatic amino acid, and one of the nine essential amino acids in the human diet [1]. In the food industry, L-Phe is the main raw material for the production of the low-calorie sweetener aspartame [2]. Microbial fermentation has become the main method for industrial production of L-Phe due to its advantages of low cost, environmental friendliness and easy process control. Due to increasing demand for high-yielding strains, rational metabolic engineering strategies were developed to redirect carbon fluxes toward L-Phe production using straightforward methods [3]. However, rational engineering has certain limitations, as they are limited to known metabolic pathways, and it is difficult to perform a comprehensive and systematic modification of the whole cell on a global level. Compared to rational design, random mutagenesis and screening is better suited for handling the complexity of the microbial system because it does not require a clear and systematic understanding of the metabolic network of the strain. Thus, it can effectively help us to direct the evolution of strains toward highly productive phenotypes through selection from a mutant library when rational modification cannot further increase the productivity of the strain [4, 5].

However, the overall metabolic profile of high-yielding strains obtained by random mutagenesis and screening is usually unclear. If we want to further improve the productivity of the desired compounds, a global understanding of the effects of metabolic and genetic modifications by quantitative omics analysis is essential to determine new potential targets. Transcriptomics enables us to investigate changes in the gene expression of production strains at the RNA level in different environments and at different time-points [6]. In addition, metabolomics enables the quantitative analysis of most metabolites in microbial fermentation [7, 8]. Although there are few reports on the comprehensive analysis of L-Phe-producing strains, the metabolic mechanisms underlying the high performance of strains producing industrial amino acids such as L-tryptophan (L-Trp) [9], L-threonine (L-Thr) [10], L-leucine (L-Leu) [11], L-valine (L-Val) [11] and L-lysine (L-Lys) [12] have been revealed by metabolomic and transcriptomic analyses. By identifying the limiting factors and potential bottlenecks limiting amino acid production, omics technology can provide effective information to further enhance the production capacity.

**Fig. 1:**
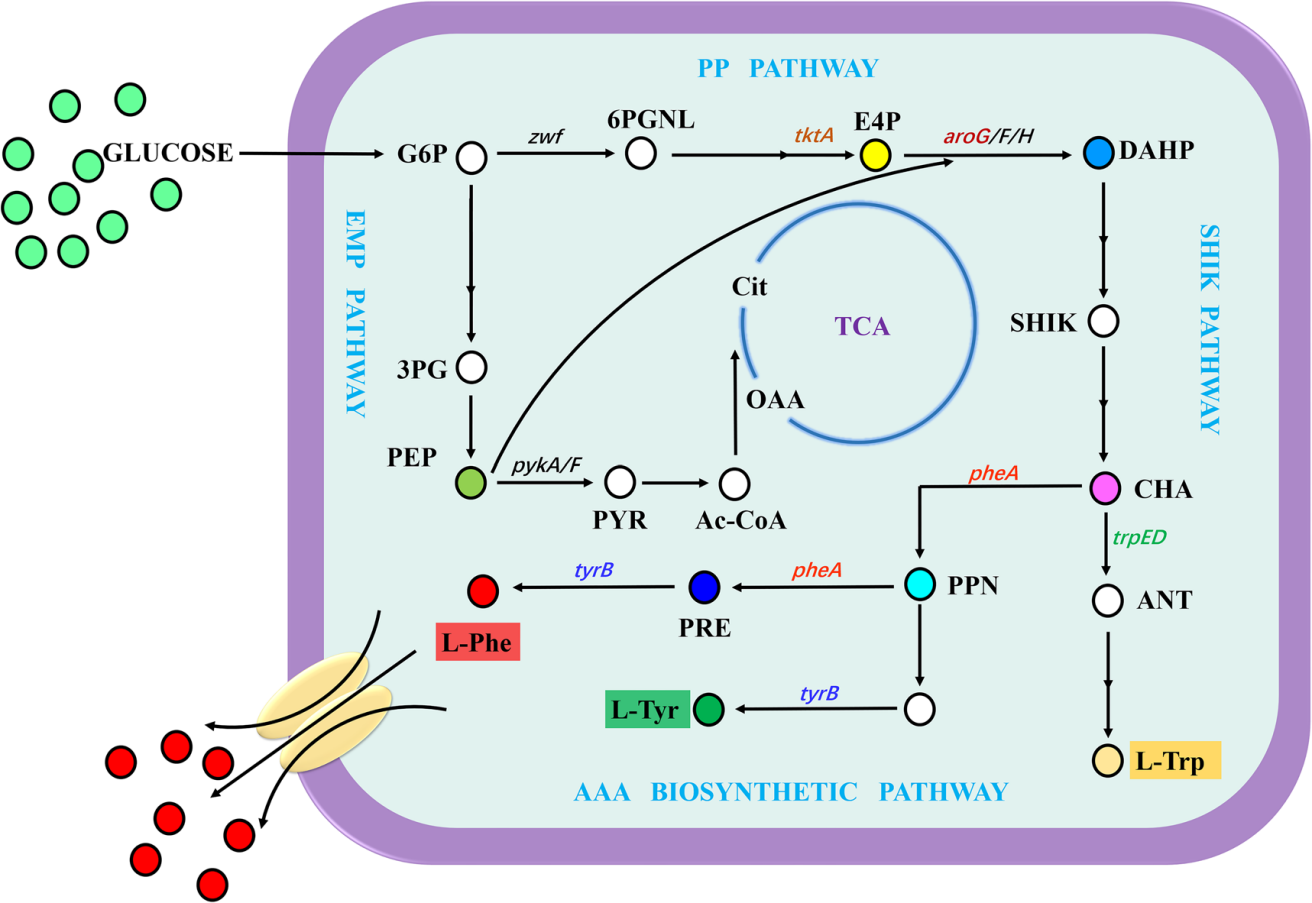
Biosynthetic pathway of L-Phe in *E. coli*. Single continuous arrows represent unique reactions catalyzed by one or more enzymes; two arrows represent two or more enzymatic reactions or incompletely characterized partial pathways. Details of the genes are listed in Additional file 1: Table S1

As shown in Fig. 1, the L-Phe biosynthesis pathway can be separated into four modules, including the central carbon metabolism (CCM), shikimate (SHIK) pathway, chorismate (CHA) pathway and L-Phe branch pathway [13, 14]. Numerous previous studies attempted to obtain L-Phe high-yielding strains by metabolic engineering or random mutagenesis and screening [3, 5], but the underlying mechanisms responsible for the increase of production, or how gene expression and metabolites change in vivo during growth and production, mostly remained unclear. In this study, a combined transcriptomic and metabolomic analysis of an L-Phe overproducing *E. coli* was performed during fed-batch culture to understand the metabolic mechanism of L-Phe overproduction during the culture process, thus providing theoretical guidance for the industrialization of L-Phe-overproducing strains in the future.

## Materials and methods

### Bacterial strain and culture conditions

The L-Phe producing strain, which was derived from *E. coli* W3110 strain and was a tyrosine auxotrophic strain. It mainly underwent multiple rounds of random mutagenesis (Additional file 2: Figure S1) followed by the overexpression of *aroF* and *pheA*^fbr^ [5, 15].

Kanamycin was added to the culture medium to a final concentration of 50 mg/L. Fed-batch fermentation was conducted as follows: (1) A single colony was seeded into 5 ml of LB liquid medium, and cultured at 37 °C and 220 rpm for 12 h; (2) The resulting initial seed culture was transferred into a 2000 ml shake flask containing 100 ml of seed culture medium (10 g/L yeast extract, 5 g/L (NH_4_)_2_SO_4_, 0.5 g/L sodium citrate, 1.3 g/L KH_2_PO_4_, 20 g/L glucose (Glc), 15 mg/L FeSO4·7H_2_O, 5 g/L MgSO_4_) at a ratio of 1:50 (v/v), and was cultured at 37 °C and 220 rpm; (3) Finally, the secondary seed culture was used to inoculate a 7-L bioreactor with 3 L of fermentation medium (5 g/L KH_2_PO_4_, 1 g/L betaine, 0.015 g/L FeSO_4_·7H_2_O, 10 g/L (NH_4_)_2_SO_4_, 5 g/L MgSO_4_, 3.3 g/L yeast extract, 0.044 g/L biotin, 20 g/L Glc) in a ratio of 1:20 (v/v) (Baoxing biology, Shanghai, China). The temperature was first kept at 33 ℃ for 12 h, after which the temperature was adjusted to 37 ℃. The air flow rate was set to 2–5 vvm, the initial stirring speed was 400 rpm, and the dissolved oxygen was maintained at approximately 40% of the atmospheric value by adjusting the rotor speed. Ammonia was added automatically to keep the pH at 7.0. When the initial sugar in the medium was exhausted, a peristaltic pump was used for automatic feeding, and the residual sugar concentration was kept below 5 g/L during the whole fermentation process. After 8 h of fermentation, samples were taken every 2 h to determine the OD_600_ and residual sugar, as well as every 4 h to determine the L-Phe concentrations.

### Analytical methods

One milliliter of fermentation broth was centrifuged at 10625 xg for 2 min. The supernatant was removed to measure the concentrations of residual sugar and L-Phe, and the bacterial cells were resuspended in 1 ml of distilled water and diluted to an appropriate value to measure the optical density at 600 nm (OD_600_). Residual sugar was determined using a biosensor analyzer (Institute of Biology, Shandong Academy of Sciences) and calibrated with 1 g/L Glc. The supernatant was passed through a 0.22 μm pore-size filter membrane before HPLC. A Zorbax Eclipse-AAA column was used for L-Phe determination. The mobile phase consisted of solvent A (40 mM Na_2_HPO_4_, pH adjusted to 7.8 with NaOH) and solvent B (ACN: MeOH: water = 9:9:2, v/v/v) at a flow rate of 2 mL/min. The specific ratios of mobile phases A and B in the injection process are 100% A (0–1.9 min), 43% A and 57% B (1.9–18.1 min), 100% B (18.1–22.3 min), and 100% A (22.3–26 min). The retention time of L-Phe was 13 min and the detection wavelength was 338 nm [16].

### Sample processing

Samples for metabolomic analysis: To detect the changes in intracellular metabolite content, samples were taken from the cultures after 8, 16, 24, 32, 40 and 48 h. Samples comprising 0.2 mL were immediately fully mixed with 1 ml 40% methanol pre-cooled to −20 ℃ by vortexing (1 s), and three parallel samples were completed within 1 min. Then, the samples were centrifuged at 10,625 xg and 4 ℃ for 2 min, the supernatant was removed, and the bacterial cells were stored at −80 ℃. Then, the cells were treated separately in 2 ml methanol pre-cooled to −20 ℃ and centrifuged for 2 min. The pellets were resuspended in 2 ml of acidic acetonitrile: water (1:1 v/v, containing 0.1% formic acid) pre-cooled to −20 ℃ and ethanol: water (3:1, v/v) pre-heated to 100 ℃. The extraction and derivation procedures were performed as described by Chang et al. [17, 18]. Finally, the obtained supernatant was freeze-dried and stored at −80 ℃.

Samples for transcriptome analysis: In the process of fed batch fermentation, transcriptome sequencing was carried out at 16, 24 and 48 h. Ten milliliters of bacterial solution were centrifuged at 4427 xg and 4 ℃ for 5 min, the supernatant was poured out, and the cells were resuspended in 10 ml phosphate buffered saline (PBS; 8 g/L NaCl, 0.2 g/L KCl, 1.42 g/L Na_2_HPO_4_, 0.27 g/L KH_2_PO_4_, pH 7.0) and washed. This step was repeated, and finally the obtained bacterial pellet was shock-frozen in liquid nitrogen and stored at -80 ℃.

### Metabolome analysis

Intracellular metabolites were analyzed using an ultra-performance liquid chromatography (UPLC) system (Nexera 30A, Shimadzu, Kyoto, Japan) coupled with a mass spectrometer (TripleTOF^™^ 5_600_, Applied Biosystem Sciex, United States) in negative electrospray ionization (ESI) mode. Most of the metabolites were separated by LC equipped with a SeQuant ZIC-HILIC column (100 × 2.1 mm, 3.5 μm, Merck, Germany). The mobile phase was composed of a gradient comprising 10 mM ammonium acetate (A) and 100% acetonitrile (B) as follows: 0–3 min, 90% B; 3–6 min, 90–60% B; 6–25 min, 60–50% B; 25–30 min, 50% B; 30–30.5 min, 50–90% B; and 30.5–38 min, 90% B, at a flow rate of 0.2 ml/min. The relative content of metabolites was normalized to the cell density.

### Library construction and sequencing

To improve the success rate of constructing the database, the samples were monitored for RNA degradation and contamination, as well as the purity, concentration and integrity of RNA. RNA degradation and contamination were monitored on a 1% agarose gel, while the other indices were respectively evaluated using a NanoDrop^®^ spectrophotometer (IMPLEN, CA, USHIK), Qubit^®^ RNA Assay Kit in Qubit^®^2.0 Fluorometer (Life Technologies, CA, USHIK) and the RNA Nano _600_0 Assay Kit of the Bioanalyzer 2100 system (Agilent Technologies, CA, USHIK). A total amount of 3 μg RNA was used as input material for each sample preparation. Sequencing libraries were generated using the NEBNext^®^ Ultra^™^ RNA Library Prep Kit for Illumina^®^ (NEB, USHIK) following the manufacturer’s recommendations, and index codes were added to attribute sequences of each sample. Then, the PCR products were purified (AMPure XP system) and library quality was assessed using the Agilent Bioanalyzer 2100 system. The results showed that the success rate of the library construction of these samples reached more than 95% (according to the criteria of DNA/RNA samples of Tsingke Biotechnology Co., Ltd.). Finally, the clustering of the index-coded samples was performed on a cBot Cluster Generation System using TruSeq PE Cluster Kit v3-cBot-HS (Illumina) according to the manufacturer’s instructions. After cluster generation, the library was sequenced on an Illumina HiSeq 2000 platform, which generated 100 bp paired-end reads.

### Transcriptome analysis

Unqualified reads in the original data were filtered to ensure the reliability of subsequent analysis results. The resulting filtered clean reads were used for subsequent analysis. In HiSeq, the reads of the comparison genome are located on the genes, and the number of reads on each gene is counted to estimate the gene expression level. To make the gene expression levels of different genes, samples and experiments comparable, FPKM (the expected number of fragments per thousand bases of the transcriptional sequence sequenced per million base pairs) was used as the gene expression level. Significantly differentially expressed genes were identified using the criteria p < 0.05 and fold change ≥ 2. At the same time, we used the cluster Profiler R package to test the statistical enrichment of differentially expressed genes in KEGG (Kyoto Encyclopedia of Genes and Genomes) pathways.

## Results

### Cultivation of L-Phe overproducing *E. coli*

**Fig. 2:**
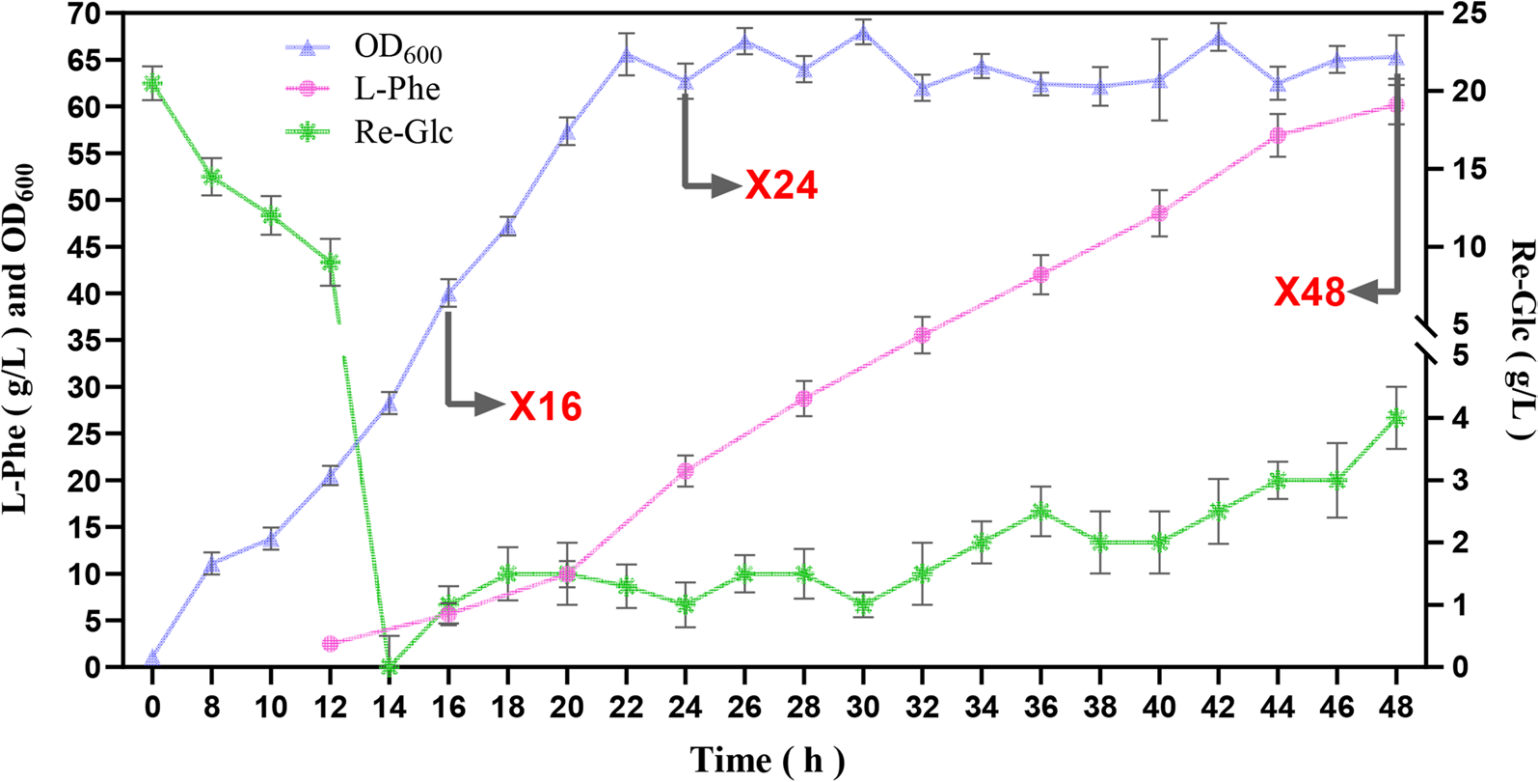
Fermentation of the L-Phe-overproducing strain in a 7-L bioreactor. Triangles indicate OD_600_, solid circles indicate the L-Phe titer, diamonds indicate the residual glucose concentration in fermentation broth. The results were comparable across the three replicates, and one was shown to illustrate the fermentation results

Starting from the wild-type *E. coli* W3110, we obtained an L-Phe-overproducing strain by ARTP mutagenesis, followed by the overexpression of *aroF* and *pheA*^fbr^. To determine the growth production ability of this strain, we performed fed-batch fermentation with intermittent Glc feeding in a 7-L bioreactor. As shown in Fig. 2, the time profile was dominated by growth in the early stages of fermentation, with the maximal rate of biomass increase reaching 5.9 h^−1^ at 16 h, and the OD_600_ reaching a maximum of 70 at 26 h. The strain began producing L-Phe after 12 h, and the productivity peaked of 2.76 g/L/h at 24 h. After 48 h of fermentation, the OD_600_ value decreased to 65.3, and the L-Phe titer reached 60 g/L, with a yield of 0.22 g/g Glc. Based on the fermentation profile, we chose samples from the most suitable time points for metabolomic and transcriptomic analyses. By evaluating changes in the content of various metabolites and the expression levels of relevant genes during the fermentation process, we aimed to identify potential targets for subsequent continuous improvement of L-Phe production.

### A large number of genes and metabolites changed dramatically during fed-batch culture of the L-Phe-overproducing *E. coli*

To better understand why this strain was capable of effectively overproducing L-Phe, a transcriptomic study was performed using samples taken at 16, 24, and 48 h of culture (named X16, X24, and X48, respectively). Additionally, metabolomic analyses were carried out using samples collected at 8, 16, 24, 32, 40, and 48 h. We observed that the relative content of metabolites and the expression levels of differentially expressed genes varied substantially over time.

**Fig. 3:**
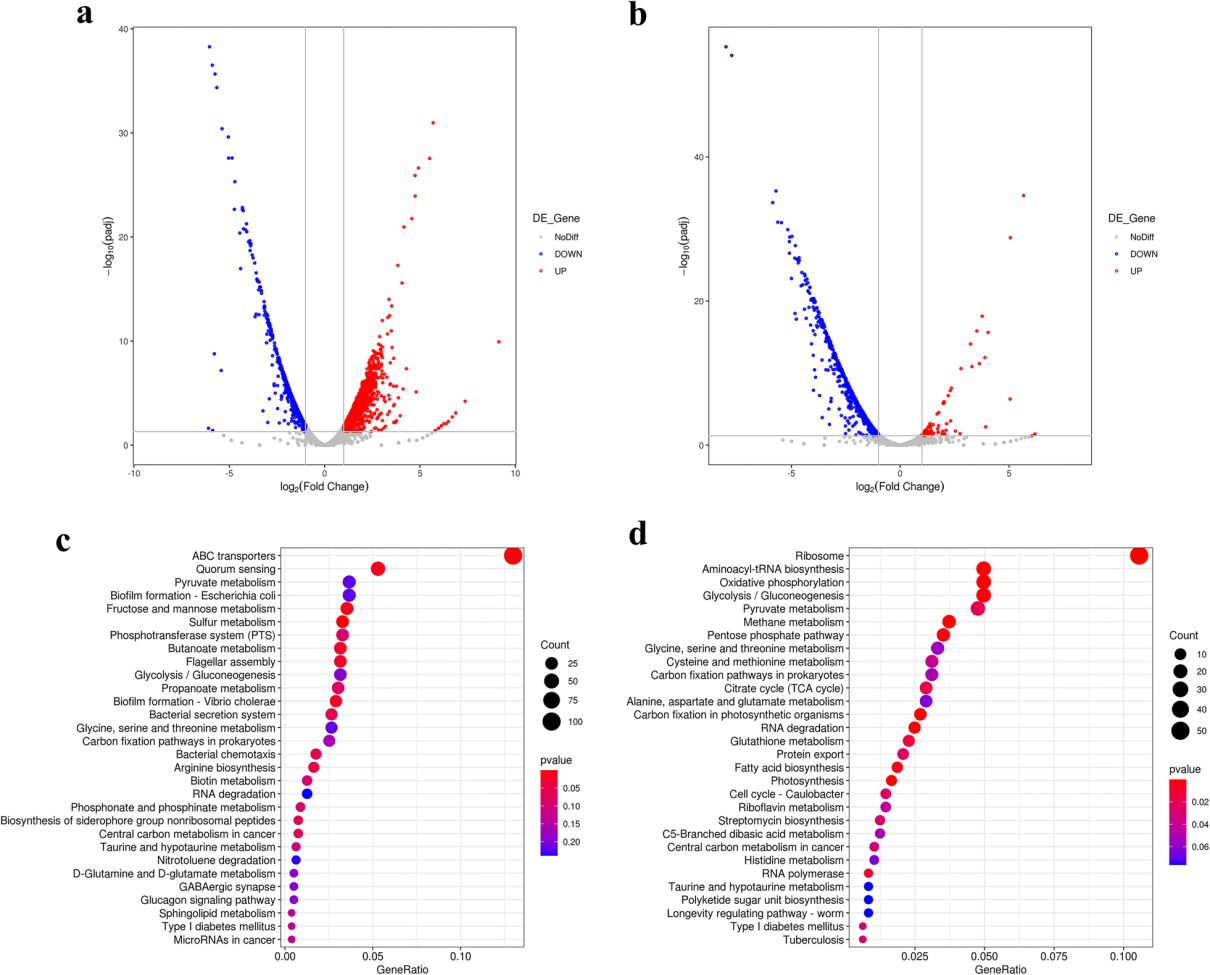
**a** and **b** Volcano plot showing the differentially expressed genes in X16 vs. X24 and X24 vs. X48. Red dots indicate upregulated genes and blue dots indicate downregulated genes (padj < 0.05 and Fold change ≥ 2). padj: Corrected P value, generally less than 0.05 is considered to indicate significant difference. **c** and **d** Kyoto Encyclopedia of Genes and Genomes (KEGG) pathway analysis of the differentially expressed genes in X16 vs. X24 and X24 vs. X48

**Fig. 4:**
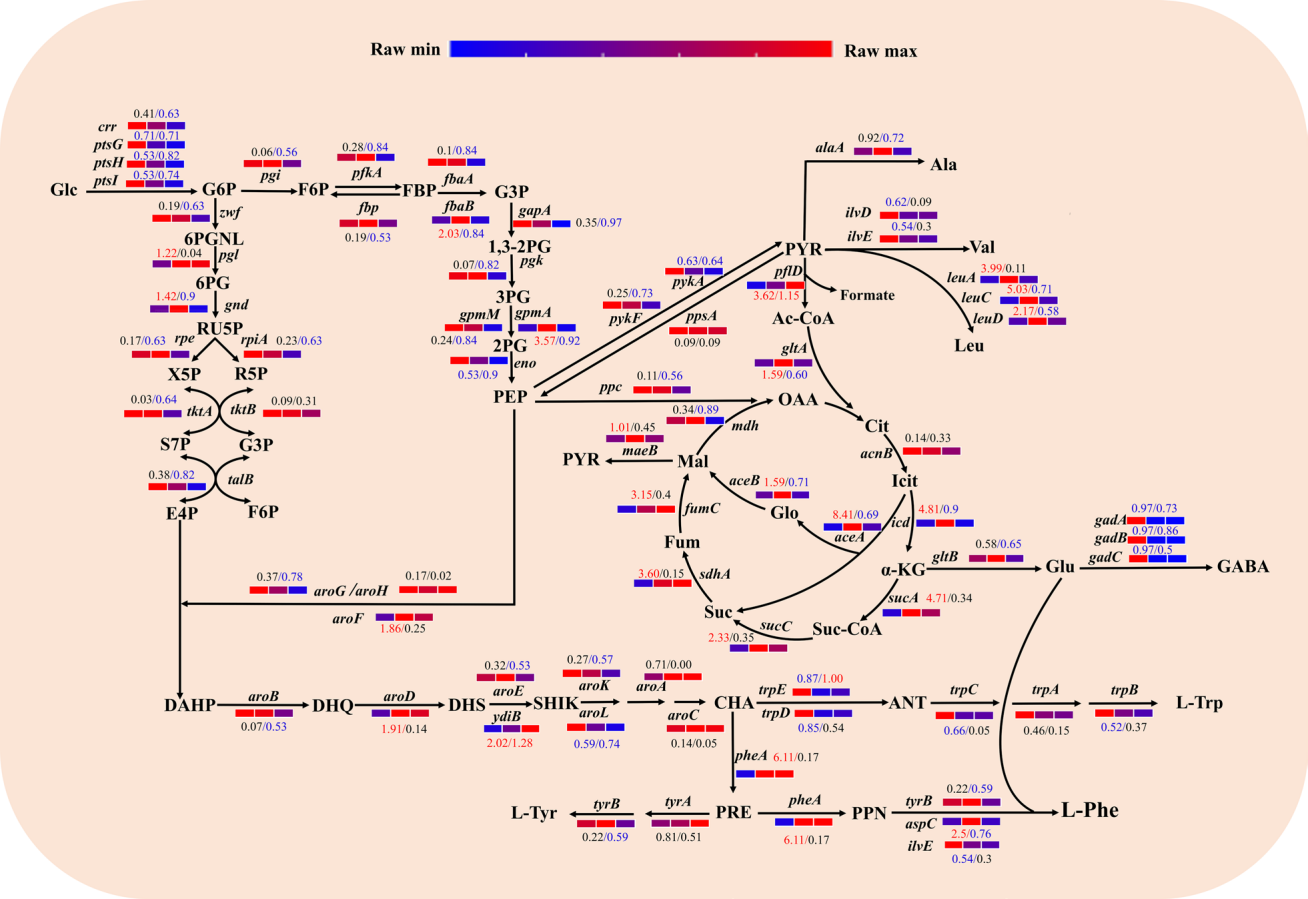
Schematic representation of the transcriptional regulation of relevant genes involved in L-Phe biosynthesis. The two numbers indicate the fold-change in X16 vs. X24 and X24 vs. X48. Red numbers indicate significant upregulation, blue numbers indicate significant downregulation, and black numbers indicate no significant difference. Bars colored from red to blue indicates higher vs. lower gene expression, respectively

The transcriptomic data revealed that 2070 genes were significantly differentially expressed in X24 compared to X16, including 1255 up and 815 downregulated genes. There were 965 significantly differentially expressed genes (DEGs) in X48 compared to X24, including 88 up and 877 downregulated genes (Fig. 3a and b). According to KEGG functional enrichment analysis, the DEGs were mainly involved in the glycolysis (EMP)/gluconeogenesis pathway, pentose phosphate (PP) pathway, pyruvate (PYR) metabolism, tricarboxylic acid cycle (TCA), CHA metabolism (Additional file 2: Figure S2), and several other metabolic pathways (Figs. 3c, d and 4), which likely directly or indirectly affect L-Phe production.

**Fig. 5:**
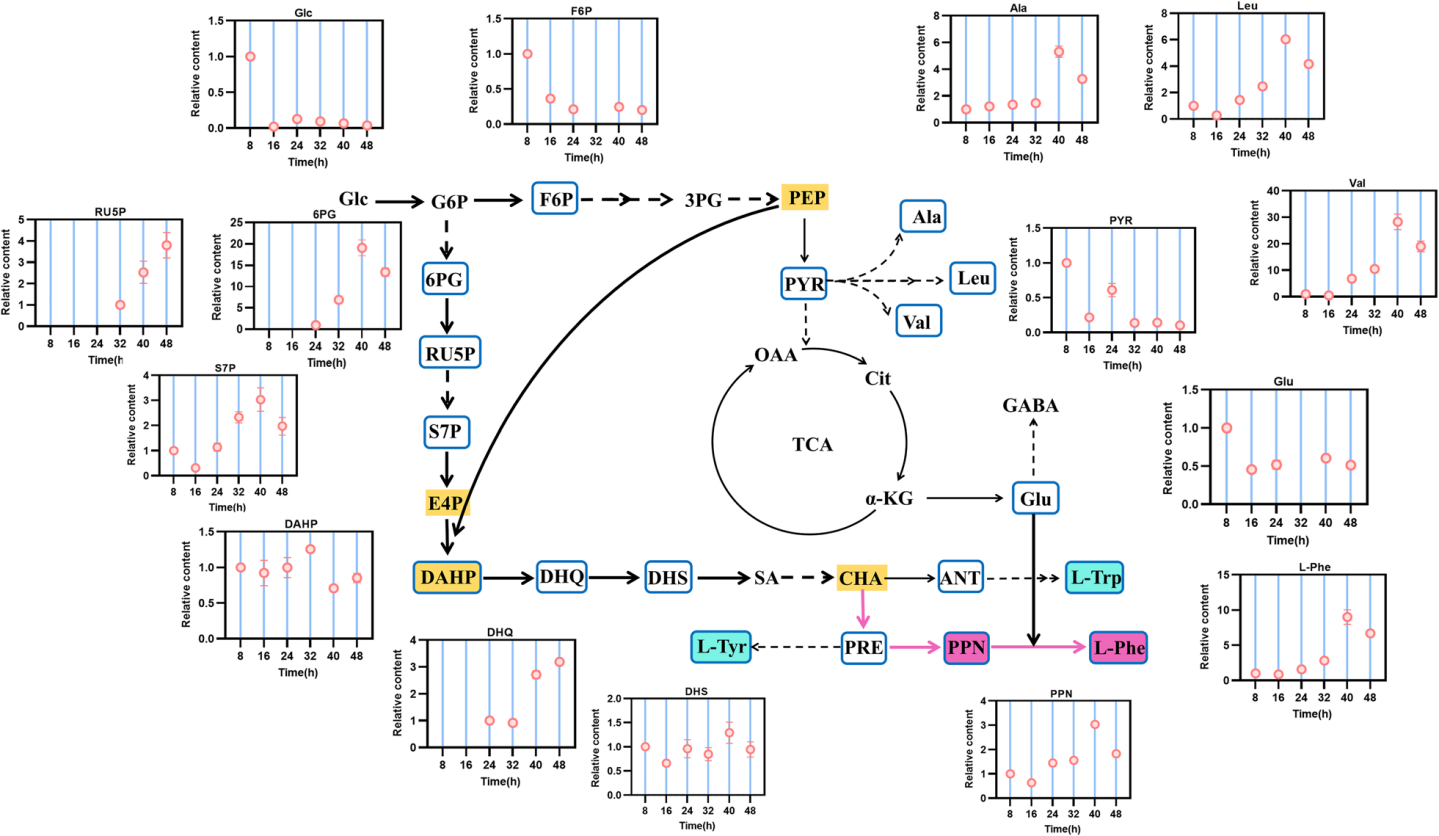
Change trends of metabolites involved in central carbon metabolism and L-Phe biosynthesis at different time points. In the graph of metabolite abundance changes, the RIA value at the time point when the cell starts to accumulate the metabolite was defined as “1” to calculate the relative value of metabolites at each later time point

Based on the metabolomic analysis, 34 differential metabolites accumulated intracellularly to varying degrees (Additional file 1: Table S3). These differential metabolites showed a substantial difference in terms of the fold change of relative intracellular abundance (RIA) and trend of change (Fig. 5). They were mostly concentrated in the EMP, PP, TCA, PYR metabolism, SHIK and CHA pathways. To better characterize this L-Phe-overproducing strain, the expression of DEGs and the RIA of metabolites in the related pathways were subjected to further analysis.

### Profiling of differentially expressed genes and metabolites in the L-Phe branch and CHA pathways

The three aromatic amino acids L-Trp, L-Tyrosine (L-Tyr) and L-Phe share all precursors and enzymes upstream of CHA, which is converted into L-Phe through three reaction steps. The *pheA* gene, which encodes chorismate mutase-prephenate dehydratase, is one of the key genes in the L-Phe biosynthesis pathway and significantly influences the production ability of the strain. The final step is catalyzed by the aminotransferase encoded by *aspC*, which transfers an amino group through a mechanism similar to that of the enzyme encoded by *tyrB* [19]. As expected, the genes *aspC* (2.5) and *pheA* (6.11) were significantly upregulated at 24 h, and the gene *tyrB* was not significantly different in X16 vs. X24 (Fig. 4). The metabolic data revealed similar trends in the RIA of PPN and L-Phe during the entire fermentation process. As shown in Fig. 5, the RIA of PPN and L-Phe remained constant and started to increase at 32 h. We speculated that the intracellular accumulation of PPN and L-Phe may be caused by the relatively low export rate. However, both RIA decreased from 40 h of cultivation, which may be related to the downregulation of the *tyrB* and *aspC* genes at 48 h, suggesting that their transcriptional levels may be a limiting factor in the production of L-Phe. It is worth nothing that the *pheA* gene maintained a high expression level in the late stage, which might explain the continuous production of L-Phe even at the end of fermentation. Overall, the data indicated that the metabolic flux tended to flow into the L-Phe branch.

In the pathway downstream of CHA, L-Trp competes with both L-Phe and L-Tyr for carbon flux. The excessive carbon flux toward the L-Tyr pathway was immediately blocked because this strain is auxotrophic for tyrosine. Significant downregulation of genes involved in the L-Trp biosynthesis pathway was observed at 24 h, including *trpE* (0.87), *trpD* (0.85), *trpC* (0.66), and *trpB* (0.52). The *trpEDCBA* genes were not significantly different in X24 vs. X48 (Fig. 4). Accordingly, we did not detect intracellular and extracellular accumulation of L-Tyr and L-Trp at 48 h. These results indicate that this strain is able to maximize the carbon flux toward L-Phe synthesis without the formation of byproducts.

### Profiling of differentially expressed genes and metabolites in the SHIK pathway

The L-Phe biosynthesis pathway begins with the condensation of PEP and E4P to form DAHP, which is then followed by a series of biochemical reactions leading to SHIK and CHA. Among the seven enzymes required for the biosynthesis of CHA from DAHP, the *aroF* (1.86), *aroD* (1.91), and *ydiB* (2.02) genes, respectively encoding DAHP synthase, 3-dehydroquinic dehydrogenase and shikimate dehydrogenase were significantly upregulated at 24 h. By contrast, the *aroL* gene (0.59) encoding shikimate kinase was significantly downregulated at 24 h. Similarly, the genes *aroG* (0.78), *aroB* (0.53), *aroE* (0.53), and *aroL* (0.74) were significantly downregulated at 48 h. However, the gene *ydiB* (1.28) remained significantly upregulated at 48 h (Fig. 4), as the cells were still producing L-Phe at this time.

According to previous reports, AroG and AroF contribute to 80% and 19% of the total DAHP synthase activity, respectively [20, 21]. Since we overexpressed *aroF*, it had a nearly 39-fold greater FPKM value at 24 h than *aroG* (21743 vs. 561), which may have a direct impact on the SHIK pathway flux (Additional file 1: Table S1). Additionally, it has been demonstrated that the formation of 3-dehydroshikimic acid (DHS) from 3-dehydroquinic acid (DHQ) is a rate-limiting step in the L-Phe biosynthetic pathway, and an increase in the expression of *aroD* facilitates the synthesis of L-Phe [3]. The downregulation of the *aroL* gene at 24 h is most likely caused by the TrpR repressor [13]. CHA is highly toxic to *E. coli*, and the downregulated of the *aroL* gene may provide a strategy to alleviate cell damage, while L-Phe is rapidly produced by attenuating its upstream metabolic flux and enhancing its downstream consumption. Accelerating CHA anabolism by may further enhance L-Phe production if done in the context of a larger CHA catabolic flow.

Different trends can be seen in the accumulation of metabolites in the SHIK pathway. Intracellular DAHP levels were essentially constant for the first 24 h, after which the RIA began to rise. Additionally, its downstream product DHQ did not exhibit any discernible changes until 32 h after it began to accumulate at 24 h. The RIA of DHS remained at nearly the same level during 8–32 h. Thus, when the cells were in the rapid L-Phe production phase, the supply of DAHP precursors was unrestricted. After 32 h, the RIA of DAHP started to decline, while DHQ accumulation abruptly increased and DHS also showed an increasing trend (Fig. 5). Consistently, the genes *aroG* (0.78), *aroB* (0.53) and *aroE* (0.53) were significantly downregulated at 48 h (Fig. 4), indicating that the downstream conversion of DAHP was decreased.

### Profiling of differentially expressed genes and metabolites in the EMP and PPP pathways

The glycolysis (EMP) and pentose phosphate (PP) pathways are two crucial components of the CCM. These pathways respectively supply phosphoenolpyruvate (PEP) and erythrose 4-phosphate (E4P), which are important precursors for L-Phe production. Half of the PEP is used by the phosphotransferase system (PTS), which catalyzes the import and concomitant phosphorylation of Glc. The three genes encoding the PTS system *ptsGIH* were significantly downregulated at the investigated timepoints, which likely contributed to the reduced loss of PEP. The majority of the EMP and PP pathway genes (*pgi*, *pfkA*, *fbp, fbaA*, *gapA*, *pgk*, *gpmM*, *pykF*, *zwf*, *rpe*, *rpiA*, and *tktA*) were significantly downregulated at 48 h but did not differ in X16 vs. X24 (Fig. 4). The intracellular metabolites of the corresponding pathways included fructose 6-phosphate (F6P), gluconate 6-phosphate (6PG), ribulose 5-phosphate (RU5P), and sedoheptulose 7-phosphate (S7P). However, the RIA of F6P in the EMP pathway gradually decreased with increasing incubation time (Fig. 5). This is likely because the rate of Glc uptake and the supply of precursors for F6P synthesis decreased with time. Additionally, the carbon from F6P still needed to be further channeled into the TCA cycle and the L-Phe synthesis pathway, which also resulted in the decreased RIA of F6P. Moreover, the expression of gene related to the EMP pathway was approximately twice as high as that of the PP pathway (Additional file 1: Table S1), and these results implied that the EMP pathway had higher carbon flux than the PP pathway, which was consistent with a recent related study [13].

The genes *fbaB* (2.03) and *gpmA* (3.57) were significantly upregulated at 24 h, which pushed more carbon flux toward PEP synthesis, even though they were not directly involved in PEP production. However, no accumulation of metabolites was detected from F6P to PEP, indicating that the EMP pathway was relatively unobstructed during the active L-Phe synthesis phase. The genes *ppsA* and *ppc* were not significantly different in X16 vs. X24, whereas *pykF* (0.63) was significantly downregulated and *aroF* (1.86) was significantly upregulated at 24 h (Fig. 4). These data suggested that most of the carbon flux from PEP was distributed to L-Phe synthesis.

The transcription of the genes *pgl* and *gnd* showed distinct regulation, as both were significantly upregulated at 24 h (1.22,1.42), but *pgl* showed no significant change in X24 vs. X48, while *gnd* (0.9) was significantly downregulated at 48 h (Fig. 4). According to the metabolite levels, the Pgl-catalyzed product 6PG, and the Gnd-catalyzed product RU5P did not begin to accumulate until 24 h and 32 h, respectively, suggesting that the pathway providing E4P was not restricted upstream of RU5P at this stage. At 48 h, the expression of the genes *rpe* (0.63), *rpiA* (0.63), *tktA* (0.64), and *talB* (0.82) was significantly downregulated. After 24 h, the TktA-generated product S7P exhibited a significantly increased RIA. The supply of E4P was able to meet the synthesis of DAHP and L-Phe according to the change in DAHP levels. We inferred that the accumulation of upstream metabolites and the downregulation of genes related to E4P may be a compensatory mechanisms that cells use to address the negative effect of excess E4P on the growth rate. Furthermore, we speculated that the DAHP synthase AroF can potentially be further modified to enhance L-Phe production.

### Profiling of differentially expressed genes and metabolites in PYR metabolism and the TCA cycle

The PEP-PYR-OAA node is a crucial junction between the TCA cycle and PYR metabolism, while the latter is the major source of the majority of byproducts that cause carbon loss. We discovered that the expression levels of genes related to Leu, Val and Ala synthesis and their RIA showed a similar trend. The genes *leuA* (3.99), *leuC* (5.03), and *leuD* (2.17) in the Leu branch pathway were significantly upregulated, while *ilvD* (0.62) and *ilvE* (0.54) in the Val branch pathway were significantly downregulated at 24 h. The *alaA* (0.92) gene in the Ala branch pathway was not significantly different in X16 vs. X24. The RIA trends of these three amino acids were almost identical, and all reached a maximum at 40 h. The accumulation of these amino acids indicated that in addition to meeting the needs of cell growth, the carbon flux was continuously leaking into the downstream PYR metabolism.

Both the *gltA* gene encoding citrate synthase (1.59), and the *pflD* gene encoding pyruvate formate lyase (3.62) were significantly upregulated at 24 h, which led to a direct flow of carbon flux into the TCA cycle, and supplied energy for cell growth. The genes *icd* (4.81), *aceA* (8.41), *aceB* (1.59), *sucA* (4.71), *sucC* (2.33), *sdhA* (3.6), and *fumC* (3.15) in the TCA cycle were all significantly upregulated at 24 h (Fig. 4). Glutamate (Glu), a precursor for the production of L-Phe that is generated from α-ketoglutarate, its RIA maintained a decreasing trend throughout the fermentation process (Fig. 5). And the Glu catabolism-related genes *gadA* (0.97), *gadB* (0.97), and *gadC* (0.97) were significantly downregulated to decrease the consumption of Glu. Thus, this strain distributed more Glu for L-Phe production in a controlled manner. In addition, we measured the extracellular Glu content (Additional file 2: Figure S3) and found that during 16–32 h, the Glu content was almost at the same level, while during 40–48 h, the Glu accumulation showed a significant upward trend, indicating that the cells were rapidly producing and consuming Glu at the same time, so the Glu supply is sufficient for the cell to produce L-Phe.

### Other genes and metabolites may be indirectly regulated in the L-Phe-overproducing *E. coli*

As previously indicated, genes related to Glc uptake were continually downregulated during the fermentation process. We discovered an intriguing phenomenon in which the genes encoding the xylose transport proteins including *xylE* (2.67), *xylF* (7.28), *xylH* (4.09), and *xylG* (7.85) were all significantly upregulated at 24 h (Table. 1). D-xylose transport proteins can take up D-xylose at the cell membrane via proton transport and promote D-Glc transport across the membrane [22]. It has been demonstrated that *E. coli* can effectively utilize other carbon sources besides Glc for the production of L-Phe. Furthermore, genes encoding other sugar transporter proteins such as *lamB* (2.46), *malE* (5.43), *malF* (7.93), *malK* (5.79), *malG* (4.56), and *glpT* (8.76) were significantly upregulated at 24 h (Table 1). According to a previous study, these transporter proteins have been linked to Glc uptake in *E. coli* [23–26]. Additionally, there was a considerable upregulation of the *galP* gene encoding the D-galactose transporter protein at 24 h (2.42). Numerous studies have demonstrated that this protein can replace the PTS system in the absorption and phosphorylation of Glc, increasing the utilization of PEP. Therefore, we speculated that GalP is likely the primary transporter responsible for Glc uptake in this strain.

**Table 1:** Transcriptional level of some other DEGs in X16 vs. X24 and X24 vs. X48

Gene name	Description	X16 vs. X24	X24 vs. X48
Sugar transportation			
*xylE*	D-xylose: H ( +) symporter	2.67	1.29
*xylF*	xylose ABC transporter periplasmic binding protein	7.28	0.51
*xylH*	xylose ABC transporter membrane subunit	4.09	1.14
*xylG*	xylose ABC transporter ATP binding subunit	7.85	1.13
*lamB*	maltose outer membrane channel	2.46	1.46
*malE*	maltose ABC transporter periplasmic binding protein	5.43	1.16
*malF*	maltose ABC transporter membrane subunit	7.93	1.08
*malK*	maltose ABC transporter ATP binding subunit	5.79	1.3
*malG*	maltose ABC transporter membrane subunit	4.56	1.15
*glpT*	sn-glycerol 3-phosphate: llmkmk phosphate antiporter	8.76	1.17
*galP*	galactose: H ( +) symporter	2.42	0.7
Phosphate transportation			
*ptsA*	putative PTS multiphosphoryl transfer protein	5.02	1.14
*phnC*	phosphonate/phosphate ABC transporter ATP binding subunit	13.37	8.93
*phnD*	phosphonate/phosphate ABC transporter periplasmic binding protein	5.07	18.55
*phnF*	putative transcriptional regulator	17.16	1.2
*phnG*	carbon-phosphorus lyase core complex subunit	13.72	-0.17
*phnI*	carbon-phosphorus lyase core complex subunit	8.61	0.31
*phnJ*	carbon-phosphorus lyase core complex subunit	7.34	2.38
*phnK*	carbon-phosphorus lyase subunit	10.15	0.28
*phnL*	methylphosphonate degradation complex subunit	11.41	0.16
*phnM*	RPnTP hydrolase	9.39	−0.08
*phnN*	ribose 1,5-bisphosphate phosphokinase	11.62	0.63
*ugpE*	sn-glycerol 3-phosphate ABC transporter membrane subunit	15.24	2.14
*ugpC*	sn-glycerol 3-phosphate ABC transporter ATP binding subunit	4.72	0.31
*pstB*	phosphate ABC transporter ATP binding subunit	1.95	0.21
*phoR*	sensor histidine kinase	2.35	1.71
*phoB*	DNA-binding transcriptional dual regulator	1.12	2.62

Intracellular phosphate metabolism is highly complex and is closely linked to CCM, which incorporates phosphate into metabolic intermediates and ATP. It has been demonstrated that altering the phosphate concentration in the medium can control the growth rate of *E. coli* [27]. As shown by transcriptome analysis, the majority of the genes found to be involved in the phosphate transporter protein system under phosphate starvation conditions were significantly upregulated at 24 h, including *ptsA*, *phnCDFGIKLMN*, *ugpE*, *ugpC*, *pstB*, *phoR* and *phoB*. The majority of these genes still showed significant upregulation at 48 h, such as *ptsA*, *phnCDFJ*, *ugpE*, *phoR* and *phoB* (Table 1). It is well known that phosphate deprivation or limitation reduces microbial growth [28]. Since the growth of this strain was steady between 24 and 48 h, a shift in carbon flux through L-Phe synthesis was triggered.

Trehalose is a common stress-induced metabolite that can create a special protective layer to shield biomolecular structures from extreme condition [29]. We discovered that the RIA of trehalose peaked at 24 h, increasing up to 70-fold from the lowest level.

## Discussion

The L-Phe-overproducing strain analyzed in this study was generated by a combination of random mutagenesis and further rational metabolic engineering. Thus, this strain evolved in a particular way under different pressures which resulted in a more intricate global metabolic network that left it unclear how L-Phe overproduction was achieved in detail. Here, we used metabolomics and transcriptomics in an attempt to provide an answer. Time-series-based transcriptomic (16, 24, and 48 h) and metabolomic (8, 16, 24, 32, 40, and 48 h) analysis were carried out in the strain. In the group comparisons X16 vs. X24 and X24 vs. X48, there were notable variations in EMP, PP, SHIK, L-Phe branch pathway, TCA cycle, and several other genes and metabolites, particularly in X16 vs. X24. Key genes involved in L-Phe biosynthesis, including *aroF*, *aroD*, *ydiB*, *pheA*, and *aspC*, were significantly upregulated at 24 h. Conversely, genes encoding enzymes that compete with L-Phe for carbon flux, such as *trpEDCBA*, were either significantly downregulated or were not altered significantly in X16 vs. X24. The other two aromatic amino acids, L-Trp and L-Tyr, were not detected throughout the fermentation process. The RIA of other metabolites, such as DAHP, DHQ, DHS and PPN, remained nearly constant until 32 h. However, Glu had an overall downward trend, which as consistent with continuous L-Phe accumulation. The high expression levels of key genes, the downregulation of the amino acid byproduct pathways, and the adequate supply of precursors can be considered the primary factors for the maximum productivity of this strain at 24 h.

The upregulation of genes related to the transport of other carbon sources, such as *xylEFHG*, *lamB*, *malEFKG*, *glpT*, and *galP*, could compensate for the downregulation of the PTS system responsible for Glc transport and phosphorylation under high-density culture conditions [3, 23, 24, 30]. Despite PTS restriction, the high productivity of this strain demonstrated that Glc uptake was not a limiting factor for L-Phe production. It has been demonstrated that phosphate limitation negatively affects biomass growth by redirecting carbon flux toward the end-product. For instance, Louise et al. [31] observed that higher SHIK production could be induced by phosphate limitation. The genes *ptsA*, *phnCDFGIKLMN*, *pstB* and *phoRB,* which were related to phosphate transport, were significantly upregulated at 24 h, and the majority of these genes remained upregulated after 48 h. We propose that the high expression of these genes may increase SHIK synthesis, which explains why the *ydiB* gene was still significantly upregulated at 48 h, resulting in a further increase of the L-Phe concentration.

Previous studies have employed a variety of strategies to increase L-Phe production in *E. coli* due to the large number of intermediates in the complex L-Phe biosynthesis pathway. These strategies included increasing the precursor supply (by knocking down *pykA* and *pykF* [32, 33], overexpressing *ppc*, *pck, tktA*, and *aroGFH* [34–36], or relieving the feedback inhibition of key enzymes (AroG and PheA) [37, 38]. Among them, enhancing precursor supply was the most popular modification strategy, typically using methods that weaken or block precursor degradation pathways while enhancing precursor synthesis pathways [39]. However, the simple increase of the precursor supply may not always produce satisfactory outcomes. The most promising strategy for boosting microbial production may be to judiciously increase conversion rates and redistribute metabolic fluxes. To further improve this L-Phe-overproducing strain, one should concentrate on improving the conversion rate rather than searching for potential bottlenecks. By replacing the PTS system, introducing the phosphoketolase XfpK, and rationally altering multiple genes at the PEP-PYR-OAA node, Xiong et al. [40] were able to redistribute fluxes of the CCM so that more carbon was effectively channeled toward the L-Trp biosynthesis pathway. The L-Phe and L-Trp synthesis pathways share serval precursors, so important recommendations from the strategy to increase L-Trp production can also be used to further increase L-Phe production.

## Conclusions

Comparative transcriptomic and metabolomic analyses were applied among different time points to clarify the mechanism underlying the overproduction of L-Phe in *E. coli*. Key metabolic properties of the examined L-Phe overproducing *E. coli* strain were the high expression of crucial genes and adequate precursor supply. In this strain, concentrating on a more rational distribution of metabolic fluxes may be a viable strategy for further improving L-Phe production. Due to the significant downregulation of the *aspC* and *tyrB* genes at 48 h, as well as *aroL* at 24 h and adequate precursor supply, we propose that metabolically engineering the SHIK and L-Phe-branch pathway may enhance L-Phe production, where t*yrB*, *aspC*, *aroL* and *aroF/*G/H are potential targets for transformation. If the activity of these enzymes or the expression level of genes is increased, it may pull more metabolic flux in the direction of L-Phe. These strategies can be used in combination with other approaches in future systems metabolic engineering studies.

## Supplementary Information

**Additional file 1: Table S1.** The significantly differentially expressed genes screened based on KEGG pathway analysis (X16 vs. X24). **Table S2.** The significantly differentially expressed genes screened based on KEGG pathway analysis (X24 vs. X48). **Table S3.** Relative abundances of intracellular metabolites at different fermentation time in an L-Phe overproducing* E. coli*.

**Additional file 2: Fig S1.** Detailed steps of strain mutagenesis. TC: Tube cultivation; ARTP: Atmospheric and room temperature plasma; FACS: Fluorescence activated cell sorting; PC: Plate cultivation; 96-WMP: 96-Well microtiter plate; MMR: Multimode microplate reader; SFC: Shake flask cultivation; HPLC: High performance liquid chromatography. **Fig S2.** Trends of transcription levels of DEGs in different metabolic pathways. a. X16 vs. X24. b. X24 vs. X48.Red indicates significantly up, green indicates significantly down, and black indicates no significant difference. **Fig S3.** The Glu determination of supernatant of fermentation broth in different periods. Experiments were conducted in triplicate and measurements are represented as the means ± S.D.

## Data Availability

All data generated and analyzed during this study are included in this manuscript and in its Additional file 1.

## Abbreviations

Glc: Glucose
G6P: Glucose-6-phosphate
F6P: Fructose-6-phosphate
FBP: Fructose-1:6-bisphosphate
G3P: Glyceraldehyde-3-phosphate
3PG: Glycerate-3-phosphate
1,3-2PG: 1,3-Diphosphoglyceric acid
2PG: Glycerate-2-phosphate
PEP: Phosphoenolpyruvate
PYR: Pyruvate
Ac-CoA: Acetyl-CoA
Cit: Citric acid
Icit: Isocitrate
Glo: Glyoxylic acid
α-KG: α-Ketoglutarate
Suc-CoA: Succinyl-CoA
Suc: Succinate
Fum: Fumarate
Mal: Malate
OAA: Oxaloacetate
6PGNL: 6-Phosphogluconolactone
6PG: 6-Phosphogluconate
RU5P: Ribulose-5-phosphate
X5P: Xylulose-5-phosphate
R5P: Ribose-5-phosphate
S7P: Sedoheptulose-7-phosphate
E4P: Erythrose-4-phosphate
DAHP: 3-Deoxy-D-arabinoheptulosonate7-phosphate
DHQ: 3-Dehydroquinate
DHS: 3-Dehydroshikimate
SHIK: Shikimate
CHA: Chorismate
PRE: Prephenate
PPN: Phenylpyruvate
L-Phe: L-Phenylalanine
ANT: Anthranilate
L-Trp: L-Tryptophan
L-Tyr: L-Tyrosine
Leu: Leucine
Val: Valine
Ala: Alanine
Glu: Glutamate
GABA: γ-Aminobutyric acid
DEGs: Differentially expressed genes
RIA: Relative intracellular abundance
CCM: Central carbon metabolism
SHIK pathway: Shikimic acid pathway
EMP: Glycolysis
PP pathway: Pentose phosphate pathway
TCA: Tricarboxylic acid cycle
ARTP: Atmospheric and room temperature plasma
KEGG: Kyoto encyclopedia of genes and genomes
