# Supplementary material for: Transcriptomics and metabolomics analysis of L-phenylalanine overproduction in Escherichia coli
Source: Microb Cell Fact. 2023 Apr 6;22:65. doi: 10.1186/s12934-023-02070-w (PMC10080781; doi:10.1186/s12934-023-02070-w)
Supplement: Supplementary file 2 — Additional file 2: Fig S1. Detailed steps of strain mutagenesis. TC: Tube cultivation; ARTP: Atmospheric and room temperature plasma; FACS: Fluorescence activated cell sorting; PC: Plate cultivation; 96-WMP: 96-Well microtiter plate; MMR: Multimode microplate reader; SFC: Shake flask cultivation; HPLC: High performance liquid chromatography. Fig S2. Trends of transcription levels of DEGs in different metabolic pathways. a. X16 vs. X24. b. X24 vs. X48.Red indicates significantly up, green indicates significantly down, and black indicates no significant difference. Fig S3. The Glu determination of supernatant of fermentation broth in different periods. Experiments were conducted in triplicate and measurements are represented as the means ± S.D. [file 12934_2023_2070_MOESM2_ESM.docx]

**Transcriptomics and metabolomics analysis of L-phenylalanine overproduction in *Escherichia coli***

Wei Sun^1,2^, Dongqin Ding^2,3,4^, Danyang Bai^2^, Yang Lin^2^, Yaru Zhu^2^, Cuiying Zhang^1^ and Dawei Zhang^2,3,4*^

* Correspondence: zhang_dw@tib.cas.cn

^1^ School of Biological Engineering, Tianjin University of Science and Technology, Tianjin 300457, China.

^2^ Tianjin Institute of Industrial Biotechnology, Chinese Academy of Sciences, Tianjin 300308, China.

^3^ National Technology Innovation Center of Synthetic Biology, Tianjin, 300308, China.

^4^ University of Chinese Academy of Sciences, Beijing 100049, China.


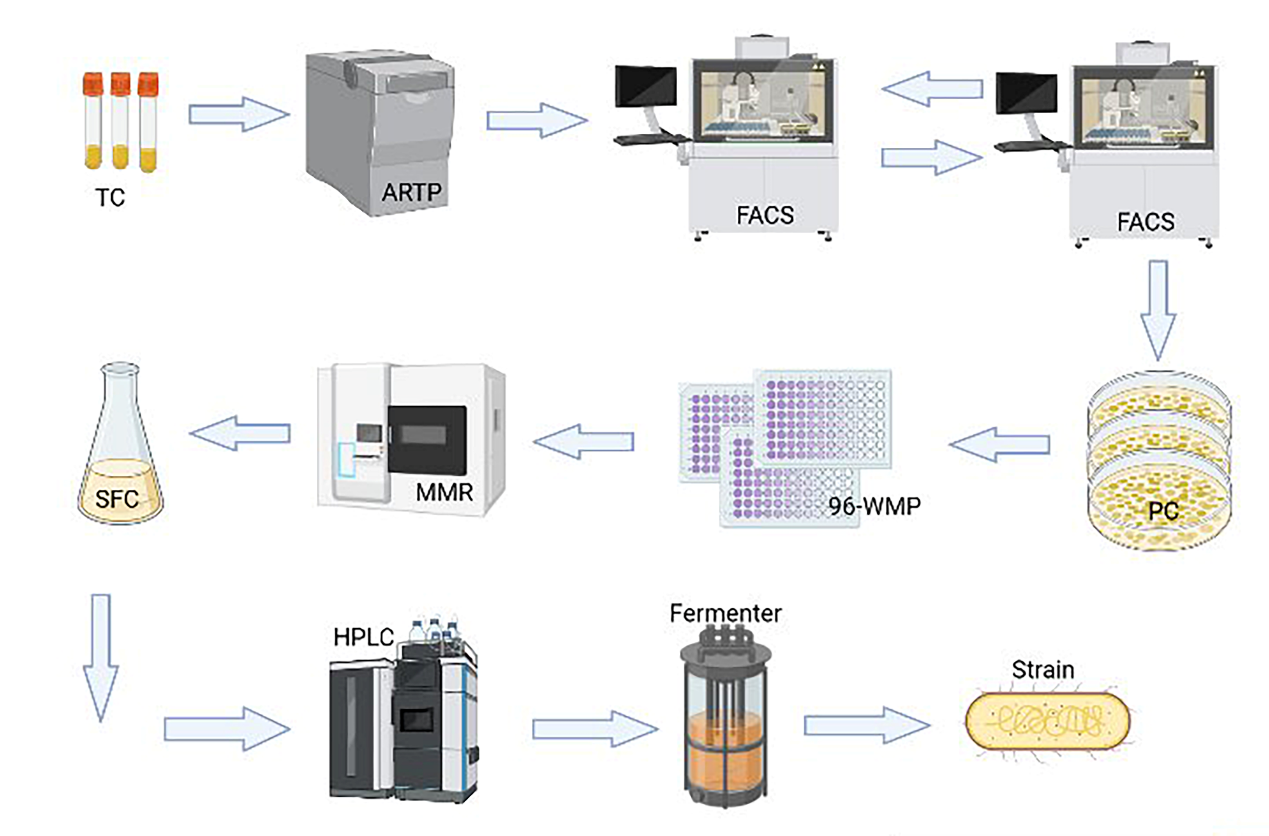


**Fig. S1** Detailed steps of strain mutagenesis. TC: Tube cultivation; ARTP: Atmospheric and room temperature plasma; FACS: Fluorescence activated cell sorting; PC: Plate cultivation; 96-WMP: 96-Well microtiter plate; MMR: Multimode microplate reader; SFC: Shake flask cultivation; HPLC: High performance liquid chromatography.

**
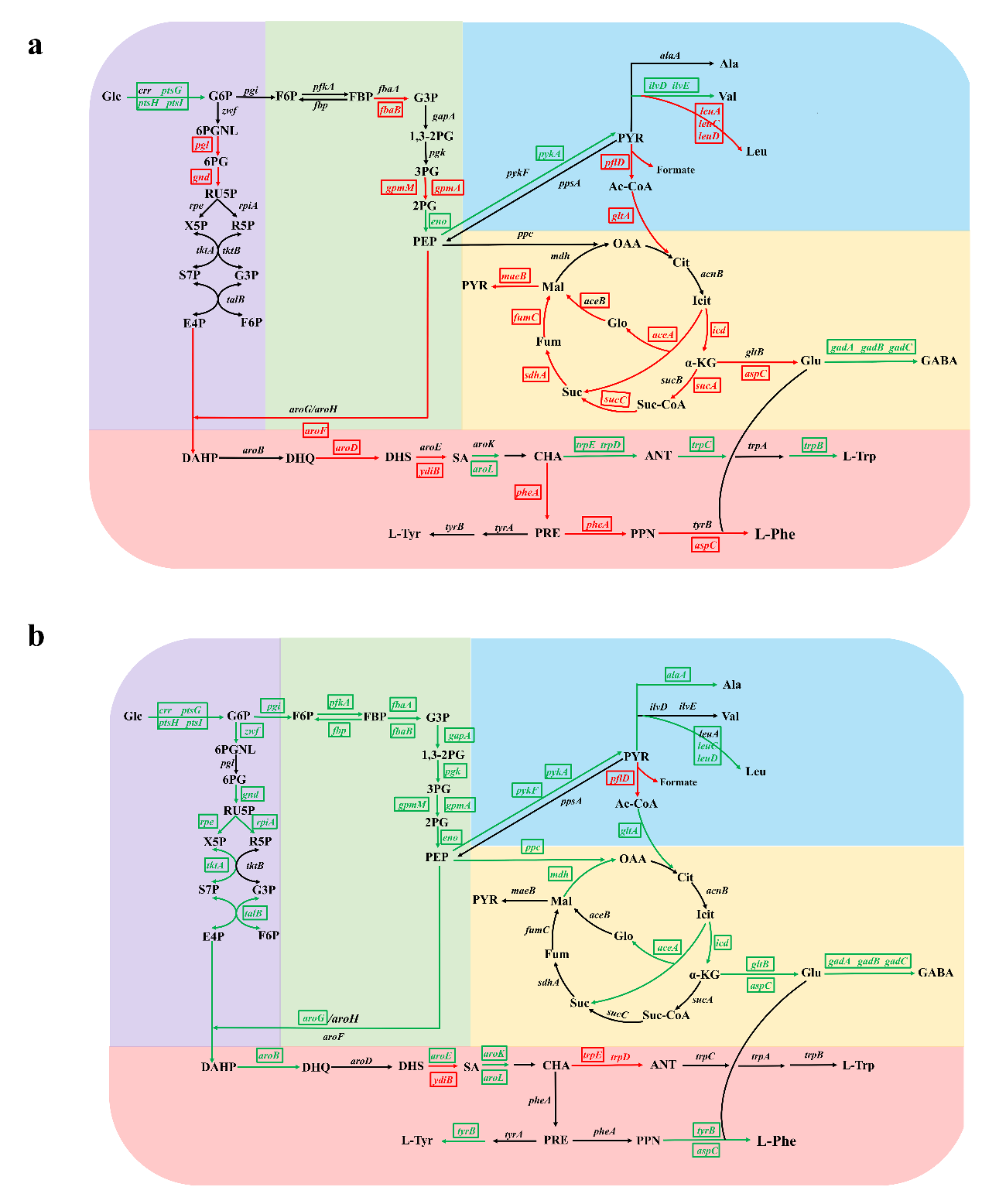
Fig. S2** Trends of transcription levels of DEGs in different metabolic pathways. **a.** X16 vs. X24. **b.** X24 vs. X48.Red indicates significantly up, green indicates significantly down, and black indicates no significant difference.

**
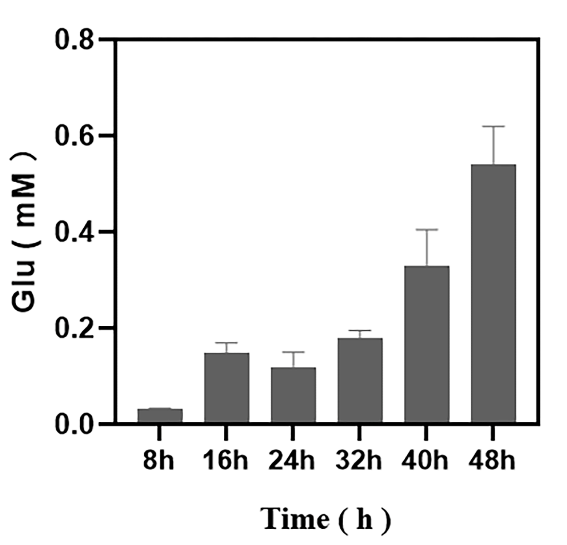
Fig. S3** The Glu determination of supernatant of fermentation broth in different periods. Experiments were conducted in triplicate and measurements are represented as the means ± S.D
